# Draft Genome Sequence of Bifidobacterium adolescentis 4-2, Isolated from Healthy Human Feces

**DOI:** 10.1128/mra.00846-21

**Published:** 2022-01-13

**Authors:** Hend Altaib, Ryo Niwa, Mayuko Abe, Tohru Suzuki

**Affiliations:** a Faculty of Applied Biological Sciences, Gifu University, Gifu, Japan; b Graduate School of Medicine, Kyoto University, Kyoto, Japan; c Graduate School of Natural Science and Technology, Gifu University, Gifu, Japan; Loyola University Chicago

## Abstract

Bifidobacterium adolescentis 4-2 was isolated from healthy human feces. Here, we report a draft genome sequence of this bacterium, which may clarify the functionality of gut microbiota-brain communication. The draft genome comprises 2.39 Mb, with an average G+C content of 59.2% and 2,028 coding DNA sequences. An operon for gamma aminobutyric acid (GABA) biosynthesis was observed in the draft genome.

## ANNOUNCEMENT

Bifidobacteria are an essential member of gut microbiota, conferring numerous health benefits (1). Recently, specific strains of *Bifidobacterium* were reported to present psychological health advantages (2). Therefore, genomic elements related to gut-brain communication have been well studied. Gamma aminobutyric acid (GABA) is an important neurotransmitter, mediating gut microbiota-brain communication (3, 4). In this study, we sequenced the strain Bifidobacterium adolescentis 4-2, isolated from the feces of healthy adult humans living in Japan, to identify gene elements which may contribute to gut microbiota-brain communication, such as the *gad* operon.

Written informed consent was obtained from the study participant, and the institutional ethics committee of Gifu University approved the study. A fecal sample was homogenized in sterile phosphate buffer saline, serially diluted, and spread onto De Man, Rogosa, and Sharpe (MRS) agar plates containing 0.05% cysteine (MRSC), followed by 48 h of entirely anaerobic incubation in a Bugbox device (Ruskinn Technology, Bridgend, UK), using a mixed gas supplement (80% N_2_, 10% CO_2_, and 10% H_2_). A single colony of the strain was subcultured several times on MRSC. DNA was extracted from a culture grown for 24 h. First, the bacterial cells were destroyed using AMR bead tubes (Advanced Microbiology Research, Gifu, Japan); then, phenol-chloroform was used for genomic DNA (gDNA) extraction.

The gDNA was used to generate a sequence library using the Next Ultra II FS DNA library prep kit for Illumina (New England BioLabs, Ipswich, MA). The fragment size distribution was analyzed using an Agilent 2100 Bioanalyzer system (Agilent Technologies, Waldron, Germany). The sample was then submitted to the Gifu University next-generation sequencing unit and sequenced in paired-end 150-bp format.

This library yielded 1,892,934 reads. The reads were assessed using FastQC v0.11.9 initially (5). Then, the adaptors and low-quality reads were trimmed using Fastp v0.20.1 in cut_front mode with a mean quality value of 30 and trim_front1 mode with a parameter of 1 bp (6). The trimmed reads were utilized for *de novo* assembly using Unicycler v0.4.8 (7). Quality check of the assembly was conducted using QUAST v5.0.5 (8). Using DFAST v1.4.0 in the Web application, gene annotation was conducted with the database (DB) type set to *Bifidobacterium* (9). CheckM v1.1.3, with the taxonomic group set to *Bifidobacterium*, was used to perform a completeness check of the draft genome on the DFAST Web application (10). Default parameters were used for all software unless otherwise specified.

The draft genome contained 33 contigs with 2,392,818 bp, with a coverage of 210×, a mean G+C content of 59.2%, an *N*_50_ value of 250,141 bp, and a total of 2,028 genes. There were a total of 4 and 58 rRNA and tRNA genes, respectively. CheckM analysis showed 99.93% completeness and 0.49% estimated contamination. Additionally, an operon for GABA biosynthesis was detected in the genome; the *gadB* (GenBank accession number LC598719) and *gadC* (LC598720) genes encoding glutamate decarboxylase and a glutamate-GABA antiporter, respectively. This finding leads us to propose that the strain be a candidate for psychobiotic applications. The availability of the whole-genome sequence of *B. adolescentis* 4-2 will allow deeper investigation into and functional analysis of genes that may contribute to gut-brain communication.

### Data availability.

The BioProject, BioSample, and DRA/SRA accession numbers for the sequence reported here are PRJDB11964, SAMD00391658, and DRR310123, respectively. The GenBank accession number of the deposited genome is BPPZ00000000.
